# Dynamic Expression of Cadherins Regulates Vocal Development in a Songbird

**DOI:** 10.1371/journal.pone.0025272

**Published:** 2011-09-20

**Authors:** Eiji Matsunaga, Kenta Suzuki, Shigeki Kato, Tohru Kurotani, Kazuto Kobayashi, Kazuo Okanoya

**Affiliations:** 1 Laboratory for Biolinguistics, RIKEN Brain Science Institute, Wako, Japan; 2 Laboratory for Symbolic Cognitive Development, RIKEN Brain Science Institute, Wako, Japan; 3 Department of Molecular Genetics, Institute of Biomedical Sciences, Fukushima Medical University, Fukushima, Japan; 4 ERATO Okanoya Emotional Information Project, JST-ERATO, Saitama, Japan; 5 Department of Life Sciences, Graduate School of Arts and Sciences, University of Tokyo, Tokyo, Japan; 6 Graduate School of Science and Engineering, Saitama University, Saitama, Japan; 7 Emotional Information Joint Research Laboratory, RIKEN Brain Science Institute, Wako, Japan; Université Pierre et Marie Curie, France

## Abstract

**Background:**

Since, similarly to humans, songbirds learn their vocalization through imitation during their juvenile stage, they have often been used as model animals to study the mechanisms of human verbal learning. Numerous anatomical and physiological studies have suggested that songbirds have a neural network called ‘song system’ specialized for vocal learning and production in their brain. However, it still remains unknown what molecular mechanisms regulate their vocal development. It has been suggested that type-II cadherins are involved in synapse formation and function. Previously, we found that type-II cadherin expressions are switched in the robust nucleus of arcopallium from *cadherin-7*-positive to *cadherin-6B*-positive during the phase from sensory to sensorimotor learning stage in a songbird, the Bengalese finch. Furthermore, in vitro analysis using cultured rat hippocampal neurons revealed that cadherin-6B enhanced and cadherin-7 suppressed the frequency of miniature excitatory postsynaptic currents via regulating dendritic spine morphology.

**Methodology/Principal Findings:**

To explore the role of cadherins in vocal development, we performed an in vivo behavioral analysis of cadherin function with lentiviral vectors. Overexpression of cadherin-7 in the juvenile and the adult stages resulted in severe defects in vocal production. In both cases, harmonic sounds typically seen in the adult Bengalese finch songs were particularly affected.

**Conclusions/Significance:**

Our results suggest that cadherins control vocal production, particularly harmonic sounds, probably by modulating neuronal morphology of the RA nucleus. It appears that the switching of cadherin expressions from sensory to sensorimotor learning stage enhances vocal production ability to make various types of vocalization that is essential for sensorimotor learning in a trial and error manner.

## Introduction

Vocal learning is a fundamental human ability. Since songbirds learn their vocalization through imitation during their juvenile stage as well, they have been used as good model animals in the study of the neural basis of human verbal learning [1]. Numerous anatomical and physiological studies have shown that songbirds have a neural network called the ‘song system’ that is specialized for vocal learning and production [2], [3]. However, although there have been important discoveries (e.g. FoxP2; [4]–[6]), it still remains largely unknown what molecular mechanisms regulate the vocal learning and production of songbirds.

Cadherins are cell adhesion molecules belonging to the cadherin superfamily that are expressed widely in a variety of tissues [7]. Among them, type-II cadherins show neural circuit-related expressions (each type-II cadherin is expressed in some restricted population of neurons that are connected with each other) [8]. In studies using mutant mice, it was proposed that type-II cadherins are involved in synaptic activity [9], [10]. However, it is not known how type-II cadherins are involved in higher brain functions at the molecular and behavioral levels.

Previously, we performed *in situ* hybridization screening and analyzed gene expression profiles of birdsong development in Bengalese finches [11]. During the vocal learning process, birds first listen to the father's song (sensory learning stage), then practice singing by themselves (sensorimotor learning stage), and finally copy the father's song. Interestingly, we found that type-II cadherin expressions are switched in the robust nucleus of arcopallium (RA) from *cadherin-7* (*Cad7*)-positive to *cadherin-6B* (*Cad6B*)-positive during the phase from sensory to sensorimotor learning [12]. Since the expression switching of cadherins is strongly related with vocal development, we hypothesized that this cadherin expression switching is involved in vocal learning and production. To address this possibility, we performed overexpression experiments of Cad7 in the RA nucleus of juvenile or adult finches with a lentiviral vector system [5], [13], and analyzed the effects on vocal production with various acoustic characteristics.

## Results

### Transient Cadherin-7 expression in the RA nucleus

**Figure 1:**
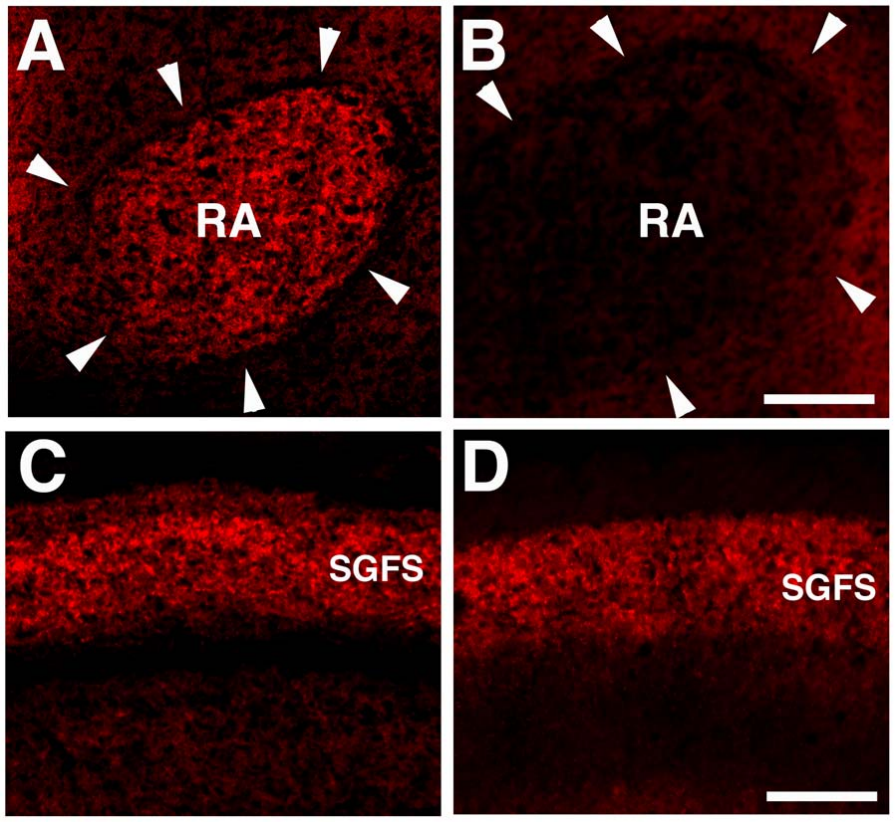
Transient Cad7 expression in RA nucleus. Immunostaining for Cad7 in the RA (A, B) and tectum (C, D) of male Bengalese finch brains (n = 5) at P30 (A, C) and P60 (B, D). Cad7 expression is disappeared by P60, even when its expression is detected in the stratum griseum et fibrosum superficiale (SGFS) layer of the tectum, as seen with *in situ* hybridization. tec: tectum. Scale bar is 500 µm.

First, we checked Cad7 expression on the protein level with a specific antibody for the Cad7 protein [14], to examine whether Cad7 mRNA expression in *in situ* hybridization studies shows a similar temporal pattern to its protein expression's. Cad7 expression was strong in almost all the cells in the RA nucleus at P30 (day 30 post-hatch) (Figure 1A; n = 5), whereas its expression almost disappeared by P60 (Figure 1B; n = 5). At the same time, Cad7 expression was seen in the tectum on the same brain sections (Figure 1C, D; n = 5). This expression pattern is very consistent with our previous *in situ* hybridization studies [12].

**Figure 2:**
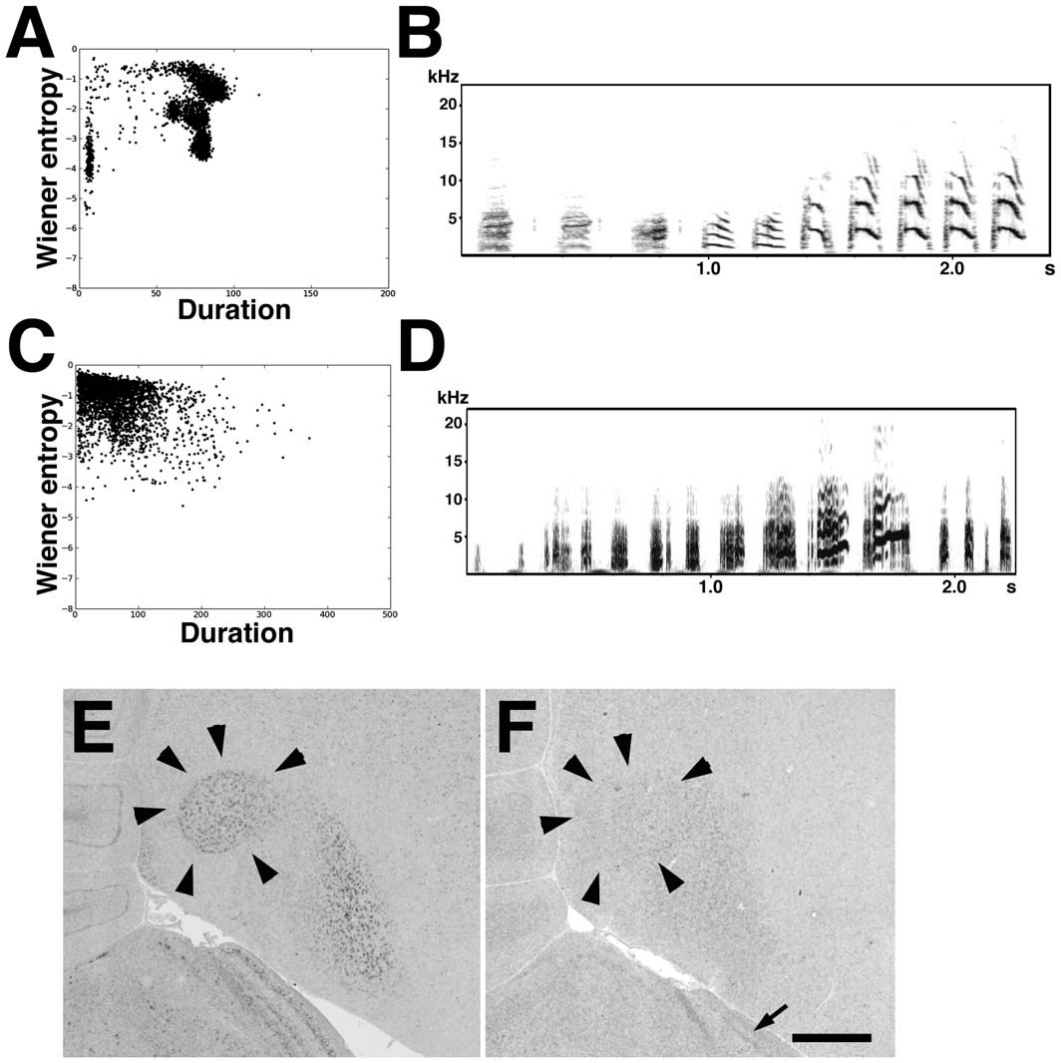
Cadherin expression is independent of breeding condition. (A, C) Two-dimensional scatterplot of 3,000 song notes (duration versus Wiener entropy) in a normally reared P120 bird (A) and an isolated P120 bird (C). This isolated bird produces ambiguous non-crystallized songs (clusters are not separated in the plot). (B, D) Sonogram of one example of song motifs of a normally reared P120 bird (B) and an isolated P120 bird (D). (E–F) *In situ* hybridization for *Cad6B* (E) and *Cad7* (F) in the same isolated P120 bird shown in panel D. Arrows indicate Cad7 expression in the SGSF layer of tectum. Although this bird produces ambiguous incomplete songs, expression of cadherins is quite similar to normally reared birds. The arrow indicates *Cad7* expression in the tectum. Scale bar is 1 mm.

To examine how cadherin expressions were affected by vocal learning itself, we examined cadherin expressions in birds reared in a vocally and socially isolated condition. A normally reared bird produces crystallized song by P120. The sound feature analysis revealed that during development sound clusters become separated in a normally reared bird (Figure 2A, B; [15]). In contrast, separated sound clusters were not seen in a bird reared in isolation, indicating that this bird produces non-crystallized songs (n = 3; Figure 2C, D). In normally reared birds, *Cad6B* expression is increased but *Cad7* expression is decreased during development [12]. As in the case of normally reared birds, up-regulation of *Cad6B* and down-regulation of *Cad7* were seen in birds under isolated conditions (Figure 2E, F). These results suggest that the change in expression is not dependent on the maturation of the songs but dependent on the maturation of neural circuits or the developmental stage.

### Cad7 overexpression by lentiviral vectors

**Figure 3:**
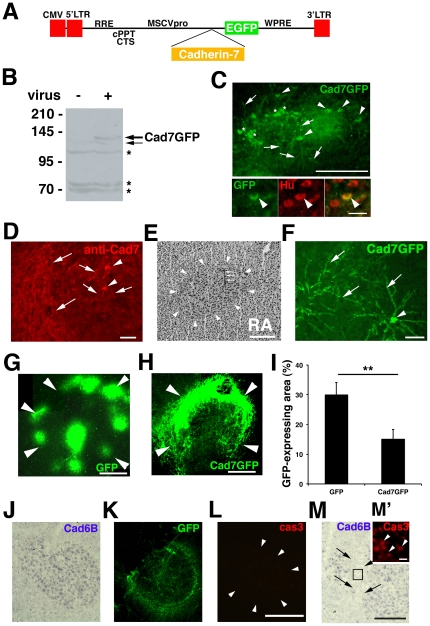
In vivo lentivirus overexpression. (A) Schematic representation of the lentivirus vector. (B) Expression analysis with anti-Cad7 antibody using Western blotting. The bands of Cad7GFP and its degraded product are seen (arrows). Asterisks indicate non-specific background as previously reported (Nakagawa and Takeichi, 1998). (C) Cad7GFP expression 2 weeks after virus injection. GFP-positive cell body (arrowheads) and fibers (arrows) and background staining (white asterisks). The virus infects Hu-positive neurons (arrowheads). (D) Cad7GFP expression is also detected in cell body (arrowheads) and fibers (arrows) by anti-Cad7 antibody 2 weeks after virus injection in an adult bird. (E) Nissl staining of the RA nucleus in a virus-injected bird at P120. Arrows indicate where the virus was injected. (F) Cad7GFP expression in a P120 bird 3 months after virus injection. Cad7GFP protein is seen in both cell body (arrowhead) and fibers (arrows). (G) GFP expression in the RA 2 weeks after virus injection. (H) Cad7GFP expression in the RA 2 weeks after virus injection. Viruses tended to be more efficiently incorporated into the peripheral region than the center of the RA nucleus. (I) Quantification of GFP-expressing areas of the RA in GFP virus- or Cad7GFP virus-injected birds. Infection efficiency of GFP expressing virus is significantly higher than Cad7GFP expressing virus in injection experiments to juvenile birds, probably because the titer of GFP virus was higher than that of Cad7GFP virus. (J–L) Cad7GFP overexpression did not affect *Cad6B* expression or apoptotic cell death in serial sections of the RA nucleus. *In situ* hybridization of *Cad6B* (J) and immunostaining for GFP (K), active caspase-3 (L) 7 days after virus injection in the adult RA. (M, M') Positive control of the active caspase-3 antibody. Active caspase-3 proteins (arrowheads) are detected in the injection site (area indicated by arrows) where tissue was injured and *Cad6B* expression is disappeared. Since this bird did not show any song defects, damage of this magnitude does not affect song production. A black square (M') indicates the position of high magnification view on the upper right corner (M). Data are shown as the mean ± SEM, with the Mann-Whitney *U*-test, * *p*<0.05 (I). Scale bars are 500 µm (E, L), 250 µm (C, M), 200 µm (D, G), 100 µm (F, H), 50 µm (C) and 10 µm (M').

To examine the role of transient Cad7 expression in the RA nucleus in vocal learning and production, we performed an overexpression experiment of the Cad7 protein. First, we made a control virus expressing enhanced green fluorescent protein (EGFP) and a virus expressing the Cad7 and the EGFP fusion protein (Cad7GFP) (Figure 3A). After we checked their expressions by western blot analysis (Figure 3B), we stereotaxically injected the GFP- or Cad7GFP-expressing virus into the RA nucleus of juvenile birds around postnatal day 20–30 (P20–30) bilaterally and examined gene expressions in vivo (Figure 3C–F). Using this system, we were able to overexpress Cad7 in neurons (Figure 3C). Cad7GFP-positive cells were seen in the RA at P120 (3 month after virus injection) (Figure 3E, F). Before analyzing the behavioral effects, we checked the efficiency of gene expressions 2 weeks after virus injection (Figure 3 G–I). We detected GFP expression in 6/7 RA injections and Cad7GFP expression in 8/16 RA injections (Figure 3G, H). Measurement of GFP-expressing areas revealed that 29.94±4.12% (n = 6) and 15.1±3.29% (n = 8) of the RA was infected by the GFP virus and the Cad7GFP virus injections, respectively (Figure 3I). One month after Cad7GFP virus injection, we still detected GFP-expression (we detected GFP expression in 5/8 injection and 13.08±3.51% (n = 5) of the RA was GFP-positive). However, it seems that overexpression by these viruses was transient and the number of virus-expressing cells was reduced by 3 months after the injections, because lentivirus vectors are able to induce exogenous gene expression without genomic integration and the efficiency of genomic integration might not be so good. Next, to verify whether virus injection induced cell death, we analyzed apoptosis with a specific antibody to the active form of caspase-3. *In situ* hybridization and immunohistochemical analysis 7 days after virus injection revealed that *Cad6B* expression was intact and no active caspase-3 positive cells were seen in the Cad7GFP-expressing region (n = 3; Figure 3J–L). On the other hand, in a different slide of the RA, *Cad6B* expression was lacking in the injection site (Figure 3M; this bird did not show any song defects). This disappearance of *Cad6B* expression might be caused by surgical injury, because *Cad6B* expression was not repressed in the Cad7GFP-expressing region (Figure 3 J, K). Active caspase-3 positive cells were only seen in the corresponding area to the injection site where *Cad6B* expression was lacking (Fig. 3 M, M'), suggesting that this antibody is able to detect cell death in the finch brain. Thus, it appears that suppression of *Cad6B* expression and cell death were not caused by Cad7GFP expression.

### Cad7 overexpression in the juvenile stage showed severe defects in vocal learning

**Figure 4:**
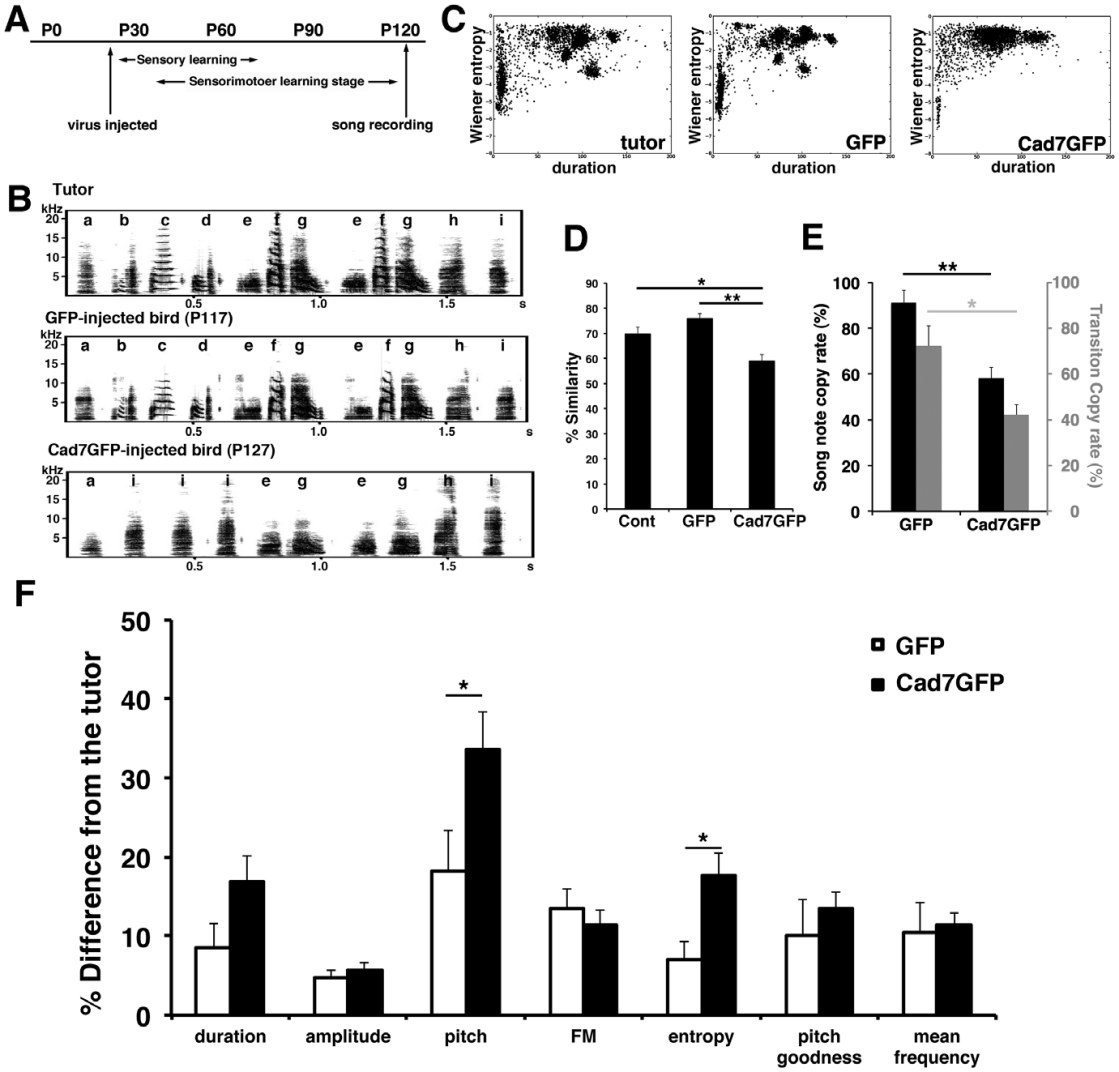
Vocal learning defects by virus injection at juvenile stage. (A) Time schedule of virus injection. (B) Typical song motifs injected birds. (C) Scatterplots of acoustic feature (duration versus Wiener entropy). (D) Song similarity and (E) Song-note and transition copy rate compared to the tutor. (F) Analysis of GFP- and Cad7GFP-bird song features. Significant differences between comparisons of tutor vs. GFP-birds and tutor vs. Cad7GFP-birds were found for pitch and entropy. FM: frequency modulation. Data are shown as the mean ± SEM. Differences were tested using the Kruskal-Wallis tests with post-hoc Games-Howell tests (D) and the Mann-Whitney *U*-test (E, F), * *p*<0.05, ** *p*<0.01.

**Figure 5:**
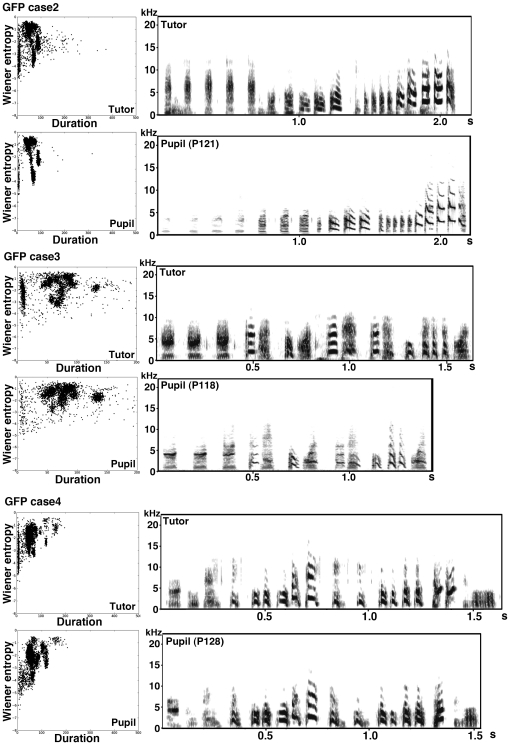
Other examples of songs of birds GFP-virus injected at juvenile stage. Sonograms of the typical song sequences of GFP-virus injected birds at postnatal 4 months, and their scatterplots of 3,000 song notes (duration versus Wiener entropy).

**Figure 6:**
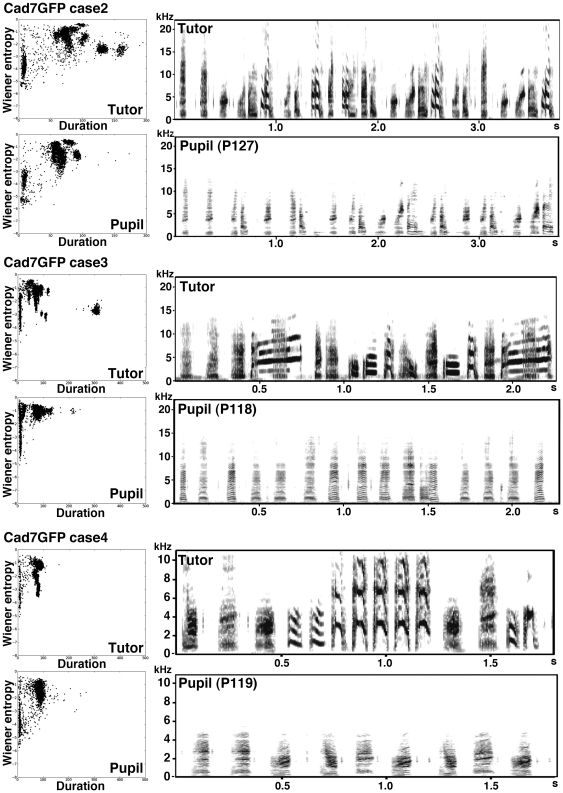
Other examples of songs of birds Cad7GFP-virus injected at juvenile stage. Sonograms of typical song sequences of Cad7GFP-virus injected birds at postnatal 4 months, and their acoustic feature analyses of 3,000 song notes with two-dimensional scatterplots (duration versus Wiener entropy).

Bengalese finch songs should crystallize by P120 [16]. To judge the effects of Cad7 overexpression on song production, we recorded songs of GFP-virus or Cad7GFP-virus injected birds around P120, 3 months after virus injection (Figure 4A). We recorded songs several times around P120, but we did not find any difference between recordings. First, we analyzed the effects on vocal production by visual inspection of the sonogram. Whereas control virus-injected birds expressing GFP (GFP-birds) successfully copied the tutor's song, Cad7GFP-expressing virus-injected birds (Cad7GFP-birds) could not copy their tutor's song, particularly harmonic song notes (Figure 4B, 5, 6, Audio S1, S2, S3). For example, in Figure 4B, the Cad7GFP-bird produces the song sequence ‘a i i i e g’, whereas the control bird produces ‘a b c d e f g’. The Cad7GFP-bird copies and uses song notes ‘a’, ‘e’, and ‘g’, as does the control, but uses ‘i’ instead of ‘b’, ‘c’, and ‘d’, perhaps by changing the sequence order. In general, as shown on Figure 4B, songs of Cad7GFP-birds have a similar temporal pattern to control birds and intact high-entropy song notes, and lack only low-entropy harmonic song notes in their song motifs. This suggests that juvenile birds injected with Cad7GFP were unable to produce only the low-entropy sounds typically seen in adult Bengalese finch songs.

Next, we examined the effects on song-note structure more precisely using the Sound Analysis Pro software (SAP; [17]). The automatic sound feature analysis revealed that compared with GFP-birds, sound clusters were not separated in Cad7GFP-birds, as seen in juvenile birds or birds reared in isolation (Figure 4C, 5, 6). Sound elements with low entropy increase with age in normally reared bird songs [15]. However, in Cad7GFP-bird songs, sound elements with low entropy were also missing (Figure 4C, 5, 6), suggesting that song development was inhibited in Cad7GFP-birds. Compared with control GFP-birds, in Cad7GFP-birds, the motif similarity (normal birds 69.75±2.63%, GFP-birds 75.92±1.87%, Cad7GFP-birds 58.75±2.71%, normal birds vs. GFP-birds: *p*>0.05, normal birds vs. Cad7GFP-birds: *p*<0.05, GFP-birds vs. Cad7GFP-birds: *p*<0.01), song-note copy rate (GFP-birds 91.10±5.51%, Cad7GFP-birds 58.06±4.67%, *p*<0.01), and sequence copy rate (GFP-birds 72.12±9.12%, Cad7GFP-birds 42.22±4.34%, *p*<0.05), were reduced (Figure 4D, E). Among various sound features, significant differences were only seen in pitch (Cad7GFP-birds 18.16±5.24%, Cad7GFP-birds 33.65±4.79% difference to tutor song, respectively, *p*<0.05), and entropy (Cad7GFP-birds 7.07±2.27%, Cad7GFP-birds 17.73±2.77% difference to tutor song, respectively, *p*<0.05) between tutor song vs. GFP-birds song and tutor song vs. Cad7GFP-birds song (Figure 4F).

**Figure 7:**
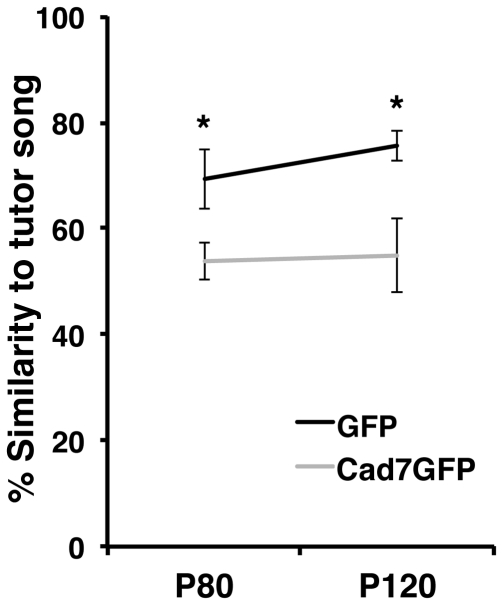
Song development of Cad7GFP virus injected birds. Developmental changes of song motif similarity of GFP-virus or Cad7GFP-virus injected bird's song to tutor's song. There is a significant difference between Cad7GFP- and GFP-virus injected birds by P80. The difference is maintained at P120. Data are shown as the mean ± SEM. Differences were tested using the Mann-Whitney *U*-test, *p<0.05.

Song defects were already seen during the vocal learning period. Analysis of songs at P80 and P120 revealed that a significant difference was detected between GFP-birds and Cad7GFP-birds by P80 and maintained at P120 (Figure 7).

### Cad7 overexpression in adult stage showed a similar defect in song production

**Figure 8:**
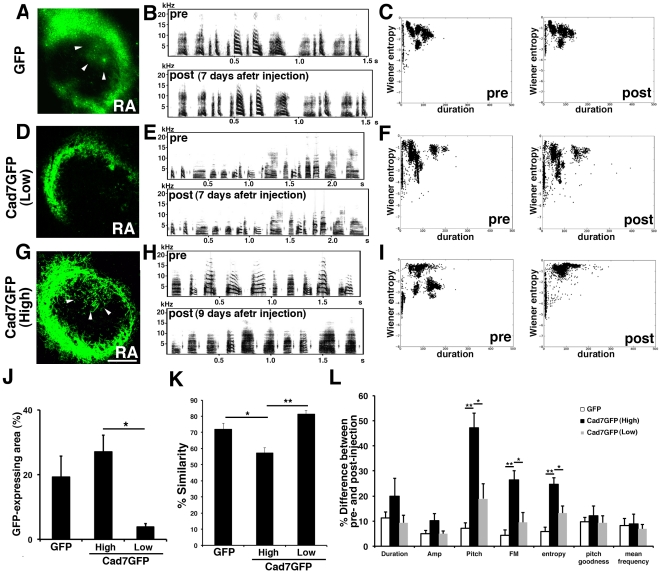
Effects on vocal production by virus injection at adult stage. GFP virus injection (A–C), low efficient Cad7GFP-virus injection (only in the peripheral region) (D–F) and high efficient Cad7GFP-virus injection (G–I) in the adult RA nucleus. (A, D, G) GFP immunostaining, (B, E, H) typical song motifs of birds before or after virus injection, and (C, F, I) acoustic feature scatterplot (duration versus Wiener entropy). (J) Efficiency of virus infection in the adult RA. For Cad7GFP injection, birds are separated into high efficiency (>10%, n = 5) and low efficiency (<10%, n = 9) injection groups. (K) Similarity score of adult GFP-, high efficiency Cad7GFP and low efficiency Cad7GFP-birds before and after virus injection. A significant difference in motif similarity was observed between high efficiency Cad7GFP-birds (n = 4) and other birds. (L) Analysis of GFP-, high efficiency Cad7GFP- and low efficiency Cad7GFP-bird song features in adult injection experiments. Comparison of the difference before and after virus injections among each group. Significant differences were found between high efficiency Cad7GFP-injected birds (n = 4) and other birds in amplitude, pitch, frequency modulation, and entropy. Data are shown as the mean ± SEM. Differences were tested using the Kruskal-Wallis tests with post-hoc Games-Howell tests, ** *p*<0.01, * *p*<0.05.

**Figure 9:**
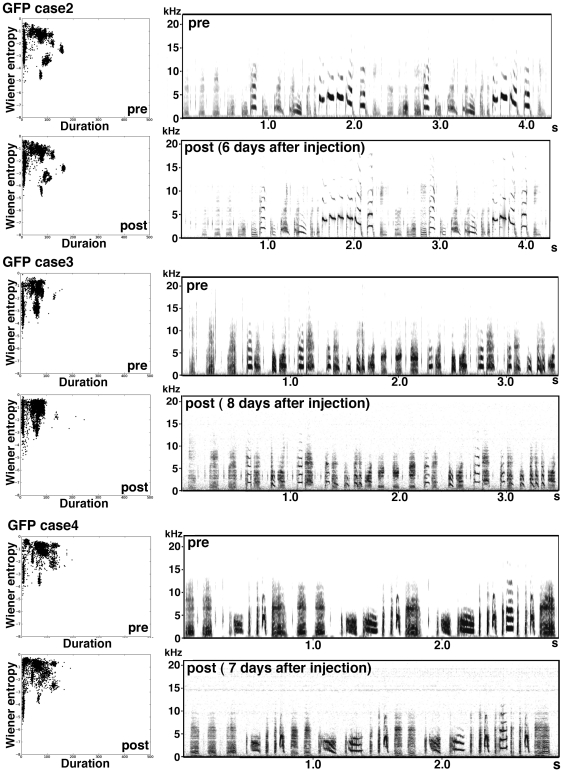
Examples of songs of birds GFP-virus injected at adult stage. Sample sonograms of the song sequences of pre- and post-GFP virus-injected birds and scatterplots of 3,000 song notes (duration versus Wiener entropy).

**Figure 10:**
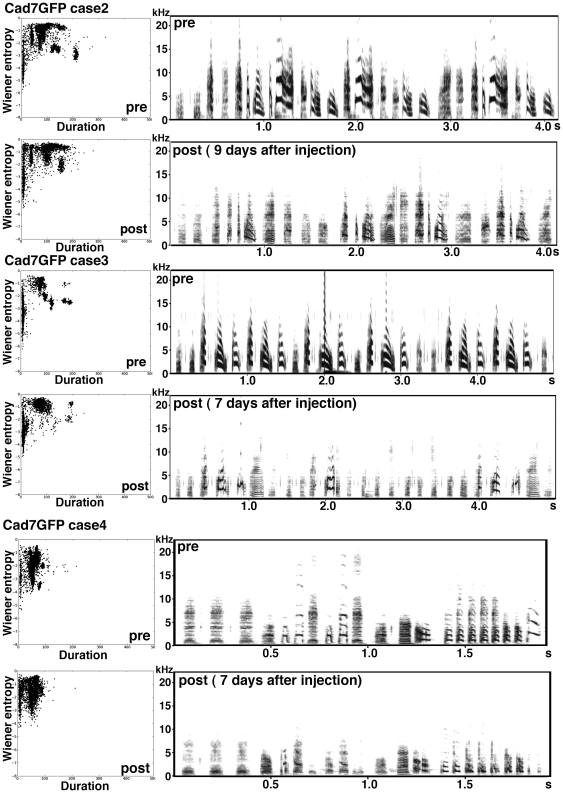
Other examples of songs of birds Cad7GFP-virus injected at adult stage. Sample sonograms of song sequences of pre- and post-Cad7GFP virus-injected birds and scatterplots of 3,000 song notes (duration versus Wiener entropy).

Next, we overexpressed Cad7 at the adult stage. Though GFP-overexpression in the RA nucleus (Figure 8A–C, 9, Audio S4, S5) or low Cad7GFP overexpression (expressed only in the peripheral region of RA nucleus) (Figure 8D–F, Audio S6, S7) showed no defects in vocal production, Cad7GFP overexpression in the RA nucleus at the adult stage also induced defects in vocal production (Figure 8G–I, 10, Audio S8, S9). As seen with overexpression at the juvenile stage, songs of Cad7GFP-birds injected at the adult stage show most severe defects in harmonic song notes (Figure 8H). After song recording, we sacrificed birds and measured the volume of the GFP-expressing region in the RA. Cad7GFP virus-injected birds were separated into two groups: a high efficiency group (High-Cad7GFP, n = 5; >10% in the sum of both hemisphere) and a low efficiency group (Low-Cad7GFP n = 9; <10%) (Figure 8J). Among them, only the high efficiency group showed significant defects in song production (Figure 8K, L). Song motif similarities between pre- and post-injection of GFP, High-Cad7GFP and Low-Cad7GFP are 71.96±3.65%, 57.12±3.25%, 81.28±2.24%, respectively (GFP vs. High-Cad7GFP: p<0.05, GFP vs. Low-Cad7GFP: *p*>0.05, High-Cad7GFP vs. Low-Cad7GFP: *p*<0.01). Sound feature analysis revealed that significant differences were seen in pitch (GFP 7.11±2.18%, High-Cad7GFP 47.17±5.77%, Low-Cad7GFP 18.91±5.93%, GFP vs. High-Cad7GFP: *p*<0.01, GFP vs. Low-Cad7GFP: *p*>0.05, High-Cad7GFP vs. Low-Cad7GFP: *p*<0.05), frequency modulation (GFP 4.33±2.16%, High-Cad7GFP 26.30±3.70%, Low-Cad7GFP 9.50±3.97%, GFP vs. High-Cad7GFP: *p*<0.01, GFP vs. Low-Cad7GFP: *p*>0.05, High-Cad7GFP vs. Low-Cad7GFP: *p*<0.05) and Wiener entropy (GFP 5.76±1.88%, High-Cad7GFP 24.65±2.66%, Low-Cad7GFP 13.14±2.96%, GFP vs. High-Cad7GFP: *p*<0.01, GFP vs. Low-Cad7GFP: *p*>0.05, High-Cad7GFP vs. Low-Cad7GFP: *p*<0.05).

## Discussion

### Harmonic sound production defects in Cad7 overexpressed birds

In this study, we injected the Cad7GFP virus at the juvenile or adult stage. In the case of juvenile injection experiments, Cad7GFP-birds tended to produce song motifs without low-entropy song notes but produced high-entropy song notes like the control birds (see Figure 4B, 4C, 6). Because we injected the viruses into the RA nucleus and sensory learning is processed in auditory areas such as the caudomedial nidopallium (NCM) and caudomedial mesopallium (CMM) [18], [19], it appears that these song defects of the Cad7GFP birds were caused by impaired vocal production. This hypothesis is supported by the results of the adult-stage injection experiments. In an adult bird, sensory learning has already been completed, and the song is crystallized. Nevertheless, Cad7GFP overexpression in adult birds changed the song sequences within only 1 week. Their songs lost harmonic notes, as in the case of the injected juveniles. It appears that Cad7 inhibits harmonic sound production in the RA nucleus during the juvenile stage, and that the change of cadherin expression from Cad7-positive to Cad6B-positive enables the birds to produce harmonic sounds typically seen in adult Bengalese finch songs. Thus, the switching of cadherin expressions may control vocal development so harmonic sounds appear in an appropriate developmental stage.

### Possible molecular mechanisms by which cadherins regulate vocal development

Very recently, it was shown that Cad6B enhances, while Cad7 inhibits, axonal branch formation in differentiating cranial motor neurons via different intracellular signaling cascades [20]. The developmental regulation of cadherin expression may play pivotal roles in various steps of neural development via the induction of various intracellular signaling pathways, including those regulating spine morphogenesis. In accordance with this, we performed *in vitro* analysis of cadherins in rat hippocampal culture neurons, and found that Cad6B increased but Cad7 decreased the number of spines and the frequency of miniature Excitatory Post Synaptic Current (mEPSC) [21]. Thus, it may be possible that the expression change of cadherins modulates electrophysiological properties to regulate vocal production in the RA nucleus. Expression change from Cad7-positive to Cad6B-positive occurred even in isolated birds, suggesting that these gene expressions are regulated by growth stage. Expression change from *Cad6B* to *Cad7* at the period from sensory to sensorimotor stage may enhance the number of excitatory synapses to increase plasticity of the RA neurons. This may facilitate the production of various types of vocalizations, particularly harmonic sounds, that are essential for sensorimotor learning in a trial and error manner. Further studies such as electrophysiological analysis with brain sections is needed to understand the detailed molecular mechanisms of cadherins in the regulation of vocal development.

## Materials and Methods

### Animals

Bengalese finches (*Lonchura striata* var. *domestica*) were obtained from a breeding colony maintained in our laboratory. The research protocols were approved by the Animal Care and Use Committee of RIKEN and conformed to National Institutes of Health Guidelines. In the normal breeding condition, each pupil was reared with his tutor. Each pupil was only isolated from his tutor during virus injection or song recording. For isolation experiments, juvenile birds were separated from their fathers by postnatal day 10 (P10), and subsequently separated from their mothers by P30. Birds were always reared in an isolated box, as described previously [22].

### Vector construction

Full-length chicken *cadherin-7* (*Cad7*) cDNA (a kind gift from Dr. Takeichi; [14]) was inserted into the *Eco*RI-*Nhe*I site of the *pCL20-MSCV-GFP* expression vector to make *pCL20-MSCV-Cad7-GFP* [23].

### Virus preparation and injection

All plasmids for lentivirus preparation were used as described previously [23]. The packaging plasmids (*pCAGkGP4.1R* and *pCAG4-RTR2*), envelop plasmid (*pCAGGS-VSV-G*), and transfer plasmid (*pCL20-MSCV-Cad7-GFP* or *pCL20-MSCV-GFP*) were transfected into HEK293T cells using the calcium phosphate precipitation method. Eighteen hours after transfection, HEK293T cells (Invitrogen) were washed with phosphate-buffered saline (PBS). Two days after transfection, the conditioned medium was collected and filtered through a 0.45-µm (pore size) cellulose filter. The viruses were precipitated by ultracentrifugation, suspended in PBS, and purified by sucrose density separation. Virus solutions were re-concentrated with the Vivaspin filtration system (Sartorius Biolabs Products) and stored at −80°C. Our viruses used here were VSVG-pseudotype that only infected anterogradely [23]. To evaluate the titers of each lentivirus (transducing units [TU]), we added lentivirus solution to HEK293T cells in a 10-cm dish, and the cell populations with GFP fluorescence were counted using flow cytometry (BD FACSAria™ II SORP; Nippon Becton Dickinson). The titers of GFP-expressing virus and Cadherin7-GFP (Cad7GFP) virus for injection were 1×10^10^ TU/ml and 1×10^8–9^ TU/ml, respectively. The tiers of GFP viruses were always higher than Cad7GFP viruses. To verify protein expression, we seeded HEK293 cells into 6-well plates, added lentivirus solution, harvested the cells, and subsequently performed Western blot analysis [24] with mouse monoclonal anti-Cad7 antibody (a kind gift from Dr. Takeichi; [14]), as described previously. The viruses were injected stereotaxically into the RA nucleus of P20–30 juvenile males or adult males aged 1–2 years anesthetized with ketamine/xylazine bilaterally. The stereotaxic coordinates for the RA were anteroposterior −0.2 to −0.4, mediolateral 1.8–2.0, and dorsoventral 1.8–2.0 for juvenile birds, and anteroposterior −0.3 to −0.5, mediolateral 2.0–2.2, and dorsoventral 2.0–2.2 for adult birds. For each injection, 400–500 nl of viral solutions were injected at several points with a glass microcapillary connected to a microinfusion pump at a constant velocity of 100 nl per minute. The sex of the juvenile birds was verified as described previously [12].

### Histological analysis, *in situ* hybridization, and immunostaining

Birds were anesthetized deeply with an intramuscular injection of sodium pentobarbital (50 mg/kg, Dainippon Seiyaku) and in some cases perfused with 4% paraformamide (PFA) containing PBS and then sacrificed. Frozen sections for immunostaining or thionine staining for neuroanatomical reference were cut serially into 20-µm-thick slices using a cryostat (Leica Microsystems). Immunostaining was performed as described previously [25]. GFP expression was detected with rabbit polyclonal anti-GFP antibody (1∶200; Molecular Probes), and apoptotic cell death was detected using polyclonal anti-activated caspase-3 (Asp 175) antibody (1∶100; Cell Signaling Technology), as described previously (Matsunaga et al., 2004). Hu expression was detected by anti-Hu antibody (A-21271) (1∶200, Molecular Probes). Cadherin-7 expression was detected with mouse monoclonal anti-cadherin-7 antibody using the same immunostaining protocol, except for slide fixation with ice-cold methanol instead of PFA. *In situ* hybridization for *cadherin-6B* and *-7* was done as described previously [12]. For quantification of virus injection efficiency, we measured the volume of the GFP-expressing region and the RA nucleus by Image J software (NIH, Bethesda, MD). We used Stat View statistical analysis software (SAS Institute, Berkeley, CA) for the statistical analysis. The analysis was done using the Kruskal-Wallis tests with post-hoc Games-Howell tests and the Mann-Whitney *U*-test, *p<0.05, **p<0.01.

### Sound recording

Because the song of a Bengalese finch is crystallized by P120, we recorded pupils' songs around P120 for the juvenile injection experiments and the isolation experiments. Some birds were also recorded around P80 to examine whether song production defects were seen during the developmental phase. For the adult injection experiment, we recorded the pre-songs about 1 week before virus injection and recorded the post-songs about 1 week after virus injection. In both cases, we recorded undirected male songs on 1–3 different days (average 200 bouts per day), but we did not find differences between the recordings. Songs were recorded in an isolated chamber at a 44.1-kHz sampling rate and 16-bit resolution with SAS Lab software (Avisoft Bioacoustics, Berlin, Germany), and the recorded sound was stored as a ‘.wav’ file in a personal computer. Before the analysis, the background noise was filtered with the GoldWave v5.22 software (GoldWave, St. John's, NF, Canada).

### Song analysis

Bengalese finch songs are composed of combinations of song notes called ‘chunks’. Unlike the zebra finch, the Bengalese finch combines song notes and chunks in different orders to make several different phrases in each bout, based on a sequential rule [16], [26], [27]. As shown in Figure 4B, in the tutor song, the song motif ‘a b c d e f g e f g h i’ is composed of song notes ‘a’, ‘b’, ‘c’, ‘d’. ‘h’, ‘i’, and chunk ‘e f g’. In this motif, the chunk ‘e f g’ is used repeatedly, whereas in different motifs, this bird produces songs such as ‘a b c d e f g h i’. During the juvenile stage, a Bengalese finch learns from his tutor how to produce each sound element and to some extent, how to order each sound element according to sequential rules.

In this study, we defined a ‘song note’ as a continuous sound element demarcated with silent intervals and the ‘song motif’ as the song sequence including all song-note types (without introductory notes). The segmentation of song notes, sound feature analysis, and similarity analysis were performed with Sound Analysis Pro2 (SAP2) software (Dr. Ofer Tchernichovski, City College of New York; [17]). We analyzed normally reared birds (n = 9), GFP-virus injected pupils (GFP pupils) (n = 8) and Cad7GFP-virus injected pupils (Cad7GFP pupils) (n = 20) in the juvenile-stage injection experiment, and GFP-virus injected birds (n = 7), highly infected Cad7GFP-virus injected birds (n = 5), and slightly infected Cad7GFP-virus injected birds (n = 9) in the adult stage injection experiments. To analyze the injection experiments in adult birds, we performed an analysis similar to that for the injection experiments in juvenile birds, as described below.

#### Sound features map

We collected 3000 song notes from each bird's song. The features of each song note were automatically calculated by the features batch module of the SAP2 software. The data was stored in MySQL tables and shown in two-dimensional scatter plots. In plots, note duration and Wiener entropy were plotted on the x-axis and the y-axis, respectively.

#### Similarity score of song motif

First, we randomly selected 30 song motifs from tutor and pupil songs. Then, we chose a pair of tutor and pupil motif and calculated the percent similarity score by comparing the five acoustic features (pitch, frequency modulation (FM), amplitude, Wiener entropy, and goodness of pitch (PG)) using the asymmetric pair-wise comparisons of the SAP2 similarity module. We repeated this calculation for 30 motif pairs to get the mean percent similarity score. For the adult injection experiment, we performed a similar analysis between pre- and post-injected bird song motifs. Statistical analysis was done using the Kruskal-Wallis tests with post-hoc Games-Howell tests, *p<0.05, **p<0.01.

#### Sound feature analysis of song notes

To evaluate the differences between the tutors' and the pupils' song-note features, we randomly selected 30 song motifs from tutors', GFP pupils', and Cad7GFP pupils' songs and calculated seven acoustic features (duration, amplitude, pitch, FM, Wiener entropy, PG and mean frequency) of each song motif by the features batch module of the SAP2 software. For each acoustic feature, we calculated the percentage differences using the following formula:
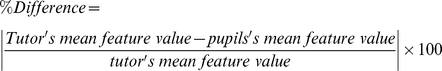
In the adult-injection experiment, we performed a similar analysis between the pre- and post-injected bird songs. To compare GFP and Cad7GFP pupils or pre- and post-injection bird songs, statistical analysis was done using the Mann-Whitney *U*-test (*p<0.05) or the Kruskal-Wallis tests with post-hoc Games-Howell tests (*p<0.05, **p<0.01).

#### Song-note copy rate

To analyze song notes, 30 undirected songs were selected randomly from tutors, GFP pupils, and Cad7GFP pupils. First, we categorized the song-note types by visual inspection and labeled each song note using unique letters, such as a, b, c… (Visual inspection and labeling were done by three independent observers to ensure validity). Then, we identified song notes copied by pupils in their own song and labeled the songs with the same letters.

The song-note copy rate was calculated as follows:
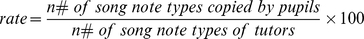
We calculated the song-note copy rate for each pupil. Statistical analysis was done using the Mann-Whitney *U*-test (**p<0.01).

#### Transition copy rate

To examine the effects of virus injection on the transition patterns of song notes, we analyzed the transition patterns in tutors' and pupils' songs. For the transition copy rate, we first marked each song note with a letter and translated song bouts into alphabet sequences. Then, we analyzed the patterns of transitions between song notes. Subsequently, we made a transition probability matrix for 30 song bouts for each individual, as described previously [28]. We measured the number of transition types as the number of transition combinations in the alphabetical orders of the 30 songs.

The transition copy rate was calculated as follows:
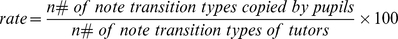
To calculate the transition copy rate, we neglected transitions with less than 5% probability and counted all other transitions. Statistical analysis was done using the Mann-Whitney *U*-test (*p<0.05).

#### Song development analysis

We used GFP-virus injected birds (n = 4) and Cad7GFP-virus injected birds (n = 5), and analyzed P80 and P120 song motifs of the same birds, and calculated the similarity score to the tutor's song. Statistical analysis between GFP-birds and Cad7GFP-birds was done using the Mann-Whitney *U*-test (*p<0.05).

## Supporting Information

Audio S1 Typical song motif of tutor bird in Figure 4B.
(MOV)

Audio S2 Typical song motif of GFP virus-injected bird in Figure 4B.
(MOV)

Audio S3 Typical song motif of Cad7GFP virus-injected bird in Figure 4B.
(MOV)

Audio S4 Typical song motif of bird before GFP virus injection into the RA nucleus in Figure 8B.
(MOV)

Audio S5 Typical song motif of bird after GFP virus injection into the RA nucleus in Figure 8B.
(MOV)

Audio S6 Typical song motif of bird before low efficient Cad7GFP virus injection into the RA nucleus in Figure 8E.
(MOV)

Audio S7 Typical song motif of bird after low efficient Cad7GFP virus injection into RA nucleus Figure 8E.
(MOV)

Audio S8 Typical song motif of bird before high efficient Cad7GFP virus injection into RA nucleus in Figure 8H.
(MOV)

Audio S9 Typical song motif of bird after high efficient Cad7GFP virus injection into RA nucleus in Figure 8H.
(MOV)
